# Physics-constrained machine learning for electrodynamics without gauge ambiguity based on Fourier transformed Maxwell’s equations

**DOI:** 10.1038/s41598-024-65650-9

**Published:** 2024-06-26

**Authors:** Christopher Leon, Alexander Scheinker

**Affiliations:** Los Alamos National Laboratory, Los Alamos, NM 87545 USA

**Keywords:** Applied physics, Computer science

## Abstract

We utilize a Fourier transformation-based representation of Maxwell’s equations to develop physics-constrained neural networks for electrodynamics without gauge ambiguity, which we label the Fourier–Helmholtz–Maxwell neural operator method. In this approach, both of Gauss’s laws and Faraday’s law are built in as hard constraints, as well as the longitudinal component of Ampère–Maxwell in Fourier space, assuming the continuity equation. An encoder–decoder network acts as a solution operator for the transverse components of the Fourier transformed vector potential, $\hat{{\textbf {A}}}_\perp ({\textbf {k}}, t)$, whose two degrees of freedom are used to predict the electromagnetic fields. This method was tested on two electron beam simulations. Among the models investigated, it was found that a U-Net architecture exhibited the best performance as it trained quicker, was more accurate and generalized better than the other architectures examined. We demonstrate that our approach is useful for solving Maxwell’s equations for the electromagnetic fields generated by intense relativistic charged particle beams and that it generalizes well to unseen test data, while being orders of magnitude quicker than conventional simulations. We show that the model can be re-trained to make highly accurate predictions in as few as 20 epochs on a previously unseen data set.

## Introduction

Electrodynamics govern a wide range of physical processes involving charged particle dynamics including universe expansion models, galactic disk formation, accelerator-based high energy X-ray light sources, achromatic metasurfaces, metasurfaces for dynamic holography, on-chip diffractive neural networks, and radiative damping of individual accelerated electrons^1–21^.

Despite widely available high performance computing, numerically calculating relativistic charged particle dynamics is still a challenge and an open area of research for large collections of particles undergoing collective effects in dynamics involving plasma turbulence^22^, space charge (SC) forces^23,24^, and coherent synchrotron radiation (CSR)^25,26^. For example, the photo-injectors of modern X-ray free electron lasers such as the LCLS, SwissFEL, and EuXFEL and plasma wakefield accelerators such as FACET-II can produce high quality intense bunches with few picosecond rms lengths of up to 2 nC charge per bunch that are accelerated and squeezed down to lengths of tens to hundreds of femtoseconds^27–32^. At low energy ($\sim$ 5 MeV) the 6D phase space $(x,y,z,p_x,p_y,p_z)$ dynamics of such bunches are strongly coupled through collective SC forces. At high energy, when particle trajectories are curved through magnetic chicanes the dynamics are coupled by CSR.

For example, a 2 nC bunch contains $N\approx \, 1.25\times 10^{10}$ electrons, for which calculating individual particle to particle SC or CSR interactions is a computationally expensive $\mathscr {O}(N^2)$ process. Conventional approaches can reduce the number of SC calculations by utilizing particle-in-cell methods, but state-of-the-art 3D CSR calculations still rely on point-to-point calculations^33^. Machine learning (ML) methods have demonstrated to be useful in non-invasive measurements and diagnostics of charged beams^34^ and in the simulations of magnetic fields through physics-informed neural networks^35^. They have also been found to speed up lattice quantum Monte Carlo simulations^36^, the design and simulation of fin field‑effect transistors^37^, the simulation of spin dynamical systems^38^ and finding the optimal ramp up for the production of Bose–Einstein condensates^39^.

Recently a new method was developed for enforcing hard physics constraints in ML, respecting physics and improving generalization^40^. In that work, 3D convolutional neural operators were used to generate the vector and scalar potential fields $\textbf{A}(\textbf{r},t)$ and $\varphi (\textbf{r},t)$, respectively, from which the electromagnetic fields were generated as $\textbf{B}(\textbf{r},t)=\nabla \times \textbf{A}(\textbf{r},t)$, $\textbf{E}(\textbf{r},t)=-\nabla \varphi (\textbf{r},t)-\partial \textbf{A}(\textbf{r},t)/\partial t$. Generating fields based on potentials as in^40^ enforces hard physics constraints, such as $\nabla \cdot \textbf{B}(\textbf{r},t)=0$ and $\nabla \times \textbf{E}+\partial \textbf{B}/ \partial t=0$, but it also adds gauge ambiguity. Building on this previous work, in this work we present a novel method for predicting electromagnetic fields, which utilizes a Fourier transformation-based representation of Maxwell’s equations and physics-constrained neural networks (PCNN) for electrodynamics without gauge ambiguity. In this approach, many of Maxwell’s equations are hard constraints: Gauss’s law for the electric and magnetic field, Faraday’s law and the longitudinal component (in Fourier space) of the Ampère–Maxwell’s law. We demonstrate this method for calculating the electromagnetic fields of intense relativistic charged particle beams.

Note: a hard constraint is one that is automatically satisfied. For example, using potentials, the condition $\nabla \cdot \textbf{B}(\textbf{r},t)=0$ automatically follows (up to numerical errors). This contrasts with a soft constraint, where one tries to guide the network to satisfy a condition by having a term in the loss function, such as $L_{\mathbf {\nabla }} = \sum _{i,j} |\nabla \cdot \textbf{B}(\textbf{r}_i,t_j)|^2$.This term encourages the network to decrease the divergence at the specified locations and times during training but offers no guarantee the network’s output will be divergenceless.

## Formalism

### Maxwell’s equations in Fourier space

The formalism that will be employed here is also used in the canonical quantization of the electromagnetic field^41^, where distinguishing between the physical degrees of freedom and the gauge redundancies is important. In the context of our study, we use it to minimize the number of fields needed to be modeled, while also ensuring that a substantial number of Maxwell’s equations are automatically satisfied as hard constraints. We start with Maxwell’s equations in position space:

1 $$\begin{aligned} \nabla \cdot \textbf{E}= \frac{\rho }{\varepsilon _0}, \quad \nabla \cdot \textbf{B}= 0, \quad \nabla \times \textbf{E}= -\frac{\partial \textbf{B}}{\partial t}, \quad \nabla \times \textbf{B}= \mu _0 \left( \textbf{J}+ \varepsilon _0 \frac{\partial \textbf{E}}{\partial t} \right) , \end{aligned}$$

where $\textbf{E}(\textbf{r},t)$, $\textbf{B}(\textbf{r},t)$, $\textbf{J}(\textbf{r},t)$, and $\rho (\textbf{r},t)$ are the time-varying electric field, magnetic field, current density, and charge density, respectively^42^. We perform a 3D spatial Fourier transform on Maxwell’s equations, which is defined for a vector field $\textbf{F}(\textbf{r},t)$ as

2 $$\begin{aligned} \hat{\textbf{F}}(\textbf{k},t) = \frac{1}{(2\pi )^{3/2}}\iiint \textbf{F}(\textbf{r},t)e^{-i\textbf{r}\cdot \textbf{k}}d^3\textbf{r}, \end{aligned}$$

and rewrite Eq. (1) (assuming $\textbf{k}\ne \textbf{0}$; see “Appendix A” for a discussion of the zero case):

3 $$\begin{aligned} i\textbf{k}\cdot \hat{\textbf{E}}= \frac{\hat{\rho }}{\varepsilon _0}, \quad i\textbf{k}\cdot \hat{\textbf{B}}=0, \quad i\textbf{k}\times \hat{\textbf{E}}= - \frac{\partial \hat{\textbf{B}}}{\partial t}, \quad i\textbf{k}\times \hat{\textbf{B}}=\mu _0\left( \hat{\textbf{J}}+\varepsilon _0\frac{\partial \hat{\textbf{E}}}{\partial t} \right) . \end{aligned}$$

For each wave vector $\textbf{k}$, a generic vector can be separated into components longitudinal and transverse to $\textbf{k}$:

4 $$\begin{aligned} \hat{{\textbf {F}}} (\textbf{k}, t) = \hat{{\textbf {F}}}_{\parallel } (\textbf{k}, t) + \hat{{\textbf {F}}}_{\perp } (\textbf{k}, t), \quad \text {such that} \quad \textbf{k}\cdot \hat{{\textbf {F}}}_{\perp } (\textbf{k}, t) = 0, \quad \textbf{k}\times \hat{{\textbf {F}}}_{\parallel } (\textbf{k}, t) = {\textbf {0}}. \end{aligned}$$

An inverse Fourier transformation of Eq. (4) will lead to a Helmholtz decomposition of ${\textbf {F}}({\textbf {r}},t)$. Gauss’s law for the electric and magnetic fields (Eq. (3)) can then be expressed as:

5 $$\begin{aligned} \hat{{\textbf {E}}}_{\parallel } (\textbf{k}, t) = -i\textbf{k}\frac{ \hat{\rho } (\textbf{k}, t) }{\varepsilon _0 |\textbf{k}|^2}, \quad \hat{{\textbf {B}}}_{\parallel } (\textbf{k}, t) = {\textbf {0}}. \end{aligned}$$

In Fourier space, the electric and magnetic fields’ dependence on the Fourier transformed scalar and vector potential, $\hat{\phi } (\textbf{k}, t)$ and $\hat{{\textbf {A}}}(\textbf{k}, t)$, is given by $\hat{{\textbf {E}}}(\textbf{k},t) = - i \textbf{k}\hat{\phi }(\textbf{k},t) - \frac{\partial \hat{{\textbf {A}}}(\textbf{k},t)}{\partial t}, \ \ \hat{{\textbf {B}}}(\textbf{k},t) = i \textbf{k}\times \hat{{\textbf {A}}}(\textbf{k},t)$. The transverse field components are:

6 $$\begin{aligned} \hat{{\textbf {E}}}_\perp (\textbf{k},t) = -\frac{\partial \hat{{\textbf {A}}}_\perp (\textbf{k},t)}{\partial t}, \quad \hat{{\textbf {B}}}_\perp (\textbf{k},t) = i \textbf{k}\times \hat{{\textbf {A}}}_\perp (\textbf{k},t). \end{aligned}$$

Using Eq. (3), Faraday’s law trivially follows:

7 $$\begin{aligned} - i\textbf{k}\times \frac{\partial \hat{\textbf{A}}_\perp (\textbf{k}, t)}{\partial t} = - \frac{\partial }{\partial t} i \textbf{k}\times \hat{\textbf{A}}_\perp (\textbf{k}, t). \end{aligned}$$

The transverse components of the Ampère–Maxwell equation become:

8 $$\begin{aligned} \frac{1}{c^2} \frac{\partial ^2 \hat{{\textbf {A}}}_\perp (\textbf{k},t) }{\partial t^2} +|\textbf{k}|^2 \hat{{\textbf {A}}}_\perp (\textbf{k},t) = \mu _0 \hat{{\textbf {J}}}_\perp (\textbf{k},t), \end{aligned}$$

while using Eq. (5) it can be shown that the longitudinal components of the Ampère–Maxwell equation is equivalent to the continuity equation. The continuity equation also relates $\hat{\textbf{J}}_\parallel$ to $\hat{\rho }$, thus only $\hat{\rho }$ and $\hat{\textbf{J}}_\perp$ need be used.

From Eqs. (5) and (6), the EM fields can then be determined by:

9 $$\begin{aligned} \hat{\textbf{E}} (\textbf{k}, t) = -i\textbf{k}\frac{ \hat{\rho } (\textbf{k}, t) }{\varepsilon _0 |\textbf{k}|^2} - \frac{\partial \hat{{\textbf {A}}}_\perp (\textbf{k},t)}{\partial t}, \quad \hat{\textbf{B}} (\textbf{k}, t) = i \textbf{k}\times \hat{{\textbf {A}}}_\perp (\textbf{k},t), \end{aligned}$$

followed by inverse Fourier transforms.

### Fourier–Helmholtz–Maxwell neural operator

**Figure 1:**
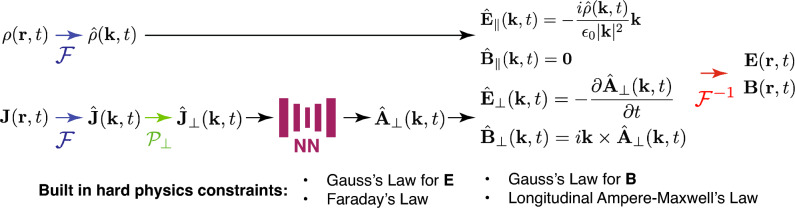
The FoHM-NO method and its built-in hard physics constraints. $\mathscr {F}$ denotes Fourier transformation, $\mathscr {P}_\perp$ is the projection of the vector field to components transverse to $\textbf{k}$, NN a neural network, and $\mathscr {F}^{-1}$ is inverse Fourier transformation.

Given the charge and current density distributions, the EM Fields can be by determined by $\hat{{\textbf {A}}}_\perp ({\textbf {k}},t)$, as per Eq. (9), and $\hat{\rho }(\textbf{k},t)$. $\hat{{\textbf {A}}}_\perp ({\textbf {k}},t)$ can be found from $\hat{{\textbf {J}}}_\perp ({\textbf {k}},t)$ via Eq. (8). From this, we propose finding the generated EM fields using the approach depicted in the diagram of Fig. 1, where the hard constraints (ones automatically obeyed) are listed. Since this approach incorporates Fourier transformations, Helmholtz decompositions, and Maxwell’s equations within a neural operator framework, we refer to it as the Fourier–Helmholtz–Maxwell neural operator (FoHM-NO) method.

In this work, we attempt to estimate the EM fields from $\rho$ and $\textbf{J}$ using the FoHM-NO method, specifically with convolutional neural networks. When applying neural networks to physics problems, prior theoretical knowledge can be leveraged to model useful quantities already identified by physicists (i.e., $\hat{{\textbf {A}}}_\perp ({\textbf {k}}, t)$), rather than the direct observables (i.e., ${\textbf {E}}({\textbf {r}}, t)$ and ${\textbf {B}}({\textbf {r}}, t)$). This has been demonstrated previously through the use of neural networks to model the Hamiltonian of classical systems^43^, the scalar and vector potentials to estimate EM fields^40^ and the stream function of an incompressible fluid’s velocity flow field^44^.

Ultimately, the FoHM-NO method exploits that the EM fields have only 2 independent degrees of freedom, as can be seen from Eq. (9) in the source-less case. On a fundamental level though, this stems from the photon being a spin-1 massless particle. One benefit of the method is that it require fewer fields than the 6 that would be needed for directly estimating both the electric and magnetic field, or the 4 necessary by using the scalar potential and the entire vector potential. With more fields to fit, there comes increasing tension between fitting the data while also satisfying physical conditions. In contrast, with this procedure by construction three of the four Maxwell’s equations (Eqs. (5) and (7)) are built-in as hard constraints. This is also the case for the longitudinal component of Ampère–Maxwell’s Law, which follows from the continuity equation and Gauss’s law for the electric field (see “Appendix B”). Working in Fourier space, meanwhile, has the advantage that spatial derivatives are traded for multiplication by wave vectors. Thus, constraints like Eq. (4) can be easier to satisfy than their spatial derivative counterparts. For a fast Fourier transform (FFT), if *N* is the total number of spatial data points, then the FFT has a time complexity of $\mathscr {O} \left( N \log N \right)$. This, along with modern GPU computing, means that moving to and fro from Fourier space can be done extremely quickly, even for very large data sets. We did not perform a temporal Fourier transform as the time length of the simulations varied. This variation would pose challenges to the networks to learn in the frequency domain. Additionally, the adoption of a 4D CNN would present computational challenges and significantly increases the number of parameters in models.

Computational EM methods done in configuration space have a notable advantage: an easy, straight-forward incorporation of spatial boundary conditions, which can be useful in solving many real-world EM problems. Here, we don’t consider any special spatial boundary conditions. For one, the simulation was just started with the initial conditions of the particles. Secondly, neural networks have been found to be useful in solving inverse PDE problems, where the problem may be ill-posed. For example, insufficient boundary conditions, a case where classical methods struggle or cannot be used^45–47^. We wish to apply our approach to inverse problems in future works.

An additional benefit of FoHM-NO is that by focusing on just $\hat{{\textbf {A}}}_\perp ({\textbf {k}}, t)$ rather than all the potential fields, the issue of gauge redundancy is bypassed. To see this, note that under a gauge transformation,

10 $$\begin{aligned} \hat{\phi } ({\textbf {k}}, t) \rightarrow \hat{\phi } ({\textbf {k}}, t) - \frac{\partial \hat{f} ({\textbf {k}}, t) }{\partial t}, \quad \hat{{\textbf {A}}} ({\textbf {k}}, t) \rightarrow \hat{{\textbf {A}}}({\textbf {k}}, t) + i {\textbf {k}} \hat{f} ({\textbf {k}}, t), \end{aligned}$$

for some scalar function $\hat{f} ({\textbf {k}}, t)$. Since the vector potential change occurs in the ${\textbf {k}}$ direction then,

11 $$\begin{aligned} \hat{{\textbf {A}}}_\parallel ({\textbf {k}}, t) \rightarrow \hat{{\textbf {A}}}_\parallel ({\textbf {k}}, t) + i {\textbf {k}} \hat{f} ({\textbf {k}}, t), \quad \hat{{\textbf {A}}}_\perp ({\textbf {k}}, t) \rightarrow \hat{{\textbf {A}}}_\perp ({\textbf {k}}, t). \end{aligned}$$

Hence, by using just $\hat{\textbf{A}}_\perp (\textbf{k}, t)$ there is no need to choose a particular gauge. The gauge invariance of $\hat{{\textbf {A}}}_\perp ({\textbf {k}}, t)$ is well known in works involving the quantization of the EM field^41,48,49^ and the spin-orbit decomposition of gauge fields^50^.

Care must be taken, though, if one were to change inertial reference frames as $\hat{\textbf{A}}_\perp (\textbf{k}, t)$, unlike the gauge-dependent $A^\mu = (\phi /c, \textbf{A})$, is not a Lorentz 4-vector. For our purposes, which is finding efficient and accurate computational methods for accelerator physics, this is not a serious issue.

The rest of this article is a proof-of-concept application of FoHM-NO for predicting the EM fields generated by relativistically charged particle beams. However, it is applicable to any EM setting where $\rho$ and $\textbf{J}$ are given, although it can also be extended to include the matter density fields in the predictions if the equations of state are used. While FoHM-NO may generally not perform as accurately as mature, state-of-the-art EM codes, the use of neural networks here gives it the advantage of having a very fast runtime. This can be useful in cases where speed is essential, such as accelerator control and diagnostics settings, where beam time can be quite limited. Alternatively, it can be used for exploratory analyses to find approximate solutions before bringing in more accurate, but slower, methods for refinement.

## Data

**Figure 2:**
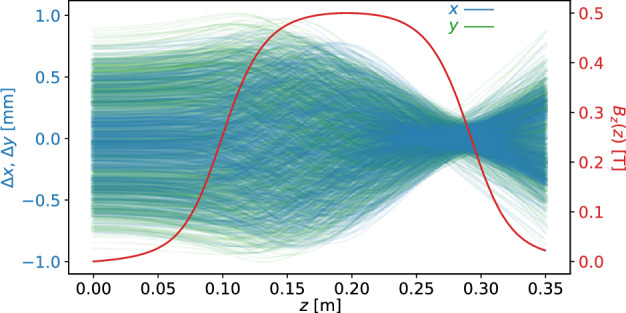
The (*x*, *y*) trajectories of 1000 random electrons within the beam are shown along with the *z*-component of the 0.5 Tesla magnetic field of the solenoid which over-compresses the beam.

**Figure 3:**
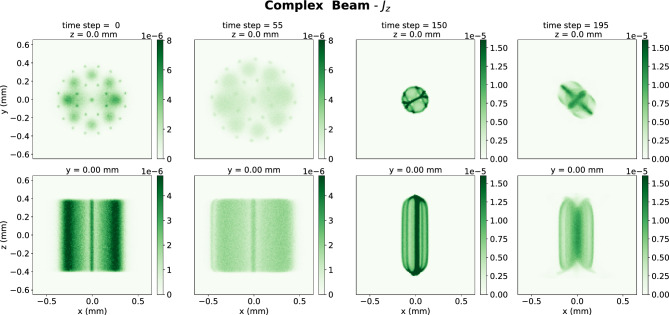
Cross-sections showing the spatial distribution of $J_z$ for the complex beam. The top row of plots shows x–y slices at z = 0 mm for different time steps. The bottom row shows x–z slices at y = 0 at the same time steps.

**Figure 4:**
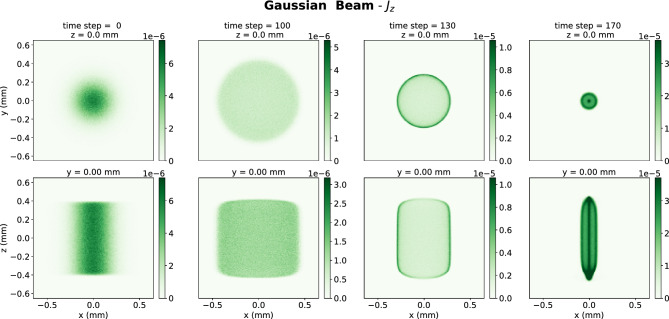
Cross-sections showing the spatial distribution of $J_z$ for the Gaussian beam. The top row of plots shows x–y slices at z = 0 mm for different time steps. The bottom row shows x–z slices at y = 0 at the same time steps.

Two simulations of electrons beams entering a solenoid magnetic fields were generated (see Fig. 2) to develop the FoHM-NO method. The first, the complex beam, was generated to train the neural networks. This complex beam was initially composed of several Gaussian bunches with spreads differing both between bunches and along the *x*, *y*, and *z* axes (see Fig. 3). The second, the Gaussian Beam, was generated to test the networks on a completely new unseen distribution which was not used for model training (see Fig. 4). This test beam starts as a Gaussian bunch before entering the magnetic field. This enabled us to test the method under very different scenarios, with the more intricate complex beam having multiple length scales in order to train the networks over a wider range of inputs. The Gaussian beam, which is much more simple, was used as a test as it more closely resembles physical beams in accelerators. All the data was generated using general particle tracer (GPT)^51,52^.

Considering the energy scales involved, GPT simulations only considered the space charge forces. For computational efficiency, GPT only registers EM fields where particles are present, creating artifacts where the EM field suddenly drop to $\textbf{0}$ where particles are absent. To address this issue, a large neutral group of particles co-moving with the beam was added to enable the registration of EM fields on the whole domain of interest without altering the motion of the electrons. Although the simulation took place on a $128\times 128 \times 128$ spatial lattice, there were artifacts where the EM fields abruptly dropped to zero on the edges (not the matter fields, however). Therefore, the EM fields were cropped to $126\times 126 \times 126$ for both beams.

Each simulations ran a 2 nC bunch of electrons with average energy 5.6 MeV moving through a 0.5 T solenoid magnetic field, first being compressed and then expanding (see Fig. 3). The space is a $128 \times 128 \times 128$ spatial grid. There were 201 time steps for the complex beam and 171 for the simple beam. The beam’s dynamics were simulated over a total time of 1.2 ns during which it traveled over a 0.35 m distance. The beam’s charge density $\rho (\textbf{r},t)$ and current density $\textbf{J}(\textbf{r},t)$ were binned as 3D histograms over cubes with 1.3 mm side lengths, as shown in Figs. 3 and 4. The physical spacing of grid were:

12 $$\begin{aligned} \Delta x = \Delta y = \Delta z = 10.2\, \upmu {\text {m}}, \quad \Delta t = 6 \,{\text {ps}}. \end{aligned}$$

To improve convergence, we first scaled the data according to standard deviation. The data had a heavy tail, so in addition a log-like transform was performed $\Lambda (x) = \text {sgn}(x) \ln \left( 1 + |x| \right)$, which has the property that larger values get greatly reduced, but smaller values stay about the same since $\Lambda (x) \approx x$ for $|x| \ll 1$.

In this work, there were no special boundary condition considerations, an external magnetic field as generated by a cylindrically symmetric solenoid was provided as input. As charged particles moved through this field their dynamics were affected by the external solenoid field and by their own self-generated electric and magnetic fields which were calculated according to Maxwell’s equations based on the charge density and current density of the charged particle beam. In this setup the accelerator beam-pipe was assumed to be of large enough radius so that image charges and currents induced in the accelerator walls did not need to be considered. In future work, boundary conditions including image charges and Maxwell’s equations on the surfaces of conductors will be incorporated into the ML model as additional hard physics constraints.

## Physics-constrained neural networks

Neural networks (NN’s) are powerful machine learning tools that, by the universal approximation theorem, can be used to approximate a function to arbitrary precision^53,54^. NN’s ability to approximate go even further by being universal approximators for operators^55^. This has been harnessed to produce approximate solutions to partial differential equations^56,57^, 3D electrodynamics^40^, and also two spatial dimensional magnetohydrodynamics simulations^58,59^.

The inclusion of physics information, constraints, or symmetries into NN be used to help guide training^60^. In some cases, these physics-incorporated NN’s can produce accurate predictions that are less computationally demanding than numerical simulations, while also being easier to develop and implement^61^. Here, we make use of a convolutional encoder–decoder architectures to make an NN that has $\hat{\textbf{J}}_{\perp }$ as input and $\hat{\textbf{A}}_{\perp }$ as output.

### Models and fit

For a relativistic beam propagating along the *z* direction, we generally have $|J_z| \gg |J_x|, |J_y|$ and consequentially it is expected that the magnetic field is predominately in the *x* and *y* direction: $|B_x|, |B_y|\gg |B_z|$. It was verified that this was indeed the case for both beam simulations used here. In fact, $B_z$ was both small and noisy, likely heavily corrupted by numerical errors. Thus, to make things more computationally efficient and increase training speed, the model was trained only using the z component of $\hat{\textbf{J}}_\perp$ to predict the z component of $\hat{\textbf{A}}_{\perp }$:

13 $$\begin{aligned} \Re (\hat{J}_{\perp , z}), \Im (\hat{J}_{\perp , z}) \rightarrow NN \rightarrow \Re (\hat{A}_{\perp , z}), \Im (\hat{A}_{\perp , z}). \end{aligned}$$

From this the transverse components of the magnetic field, $\hat{B}_x$ and $\hat{B}_y$, are obtained via $\hat{\textbf{B}} = i \textbf{k}\times ( \hat{A}_{\perp , z} \hat{\textbf{z}})$. The models were trained on the first 85% of the complex beam run, which is the training set. The remainder, validation set, was used for hyperparameter tuning. The entire Gaussian Beam data was used as test data.

#### Architectures

**Figure 5:**
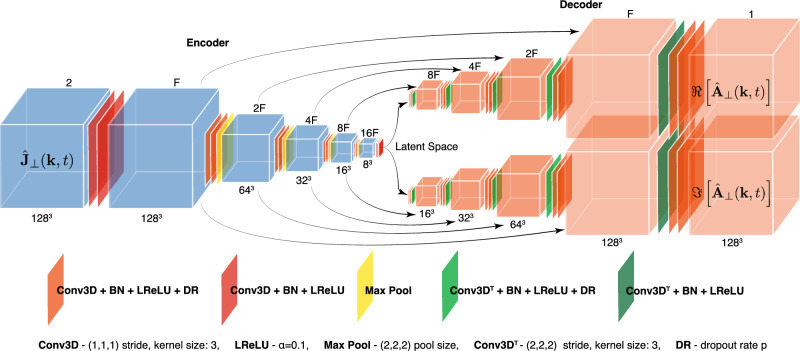
The adapted U-Net architecture is shown. For the number of starting filters we took $F=16$. The skip connection concatenates two feature maps of the same dimensions. The CNN architectures were very similar, with the skip connections removed and the number of filters in each layer changed with CNN-small-4 having filter numbers 16, 32, 64, 128, 64, 4, 64, 64, 32, 16 between the inputs and outputs. Having a double convolution layer before the in the input and output improved results. The output here is also cropped to $126\times 126 \times 126$ (see text).

Different architectures for the neural network were explored to optimize performance. The first models trained had a CNN encoder–decoder architecture design, which is depicted in Fig. 5 (once the skip connections are removed). This was chosen as the reduction and reconstruction from the small latent space acts as an effective regularizer. In addition, it was successful in a previous beam simulation task^40^. The encoder compresses the input $\hat{J}_{\perp , z}$ to the latent space via various functional compositions, represented as layers. From there, two decoder arms are applied to the latent space, one producing $\Re (\hat{A}_{\perp , z})$ and the other $\Im (\hat{A}_{\perp , z})$. Empirically, this bifurcated structure produced better results than having a single decoder that gave the both real and imaginary components of $\hat{A}_{\perp , z}$ as output.

We made two models following Fig. 5 differing by the number of filters in the latent space, 4 and 6 filters, which we label CNN-small-4 and CNN-small-6, respectively. Also, we used an architecture similar to CNN-small-4, but with more filters in the hidden layers and thus more parameters, dubbed here CNN-large. We also investigated the adaption of an U-Net encoder–decoder architecture^62^, visually depicted in Fig. 5, which we call U-Net. U-Nets have found applications in scientific computation^63,64^ and computer vision. One limitation of the CNN encoder–decoder architecture in Fig. 5 is that information of the input gets ‘forgotten’ as it’s compressed to the latent space. The U-Net addresses this by adding ‘skipped connections’ between hidden layers of the encoder and decode that share the same dimensions. These connections concatenate the feature maps, allowing the decoder to pull information from the encoder and generally reproduce finer details more accurately than CNN architectures without skipped connections. The skipped connections are also useful during the training process by allowing for more gradient flow, thus can act as an effective tool against vanishing gradients and can speed up training.

#### Fits

An $L_2$ penalty for the weights and dropout with $p=0.05$ were used for regularization. All the NN’s were created, trained and tested using Tensorflow^65^, with training being done using the Adam optimizer^66^ with early stopping based on validation loss. All the machine learning training was done on a Dell Precision 7920 with two Nvidia RTX A6000 GPU’s with 48 GB VRAM each. A summary of the four architectures with a description can be found in Table 1.

**Table 1:** Investigated architectures are arranged in descending order based on total number of parameters.

Model	Description
CNN-small-4	CNN encoder–decoder, with 4 filters in the $8\times 8\times 8$ latent space
CNN-small-6	CNN encoder–decoder, with 6 filters in the $8\times 8\times 8$ latent space
U-Net	Encoder–decoder with skipped connection, with $F = 16$
CNN-large	The architecture is similar to CNN-small-4, but with more filters in the hidden layers

#### Cost function and metric

NNs were trained to minimize mean squared error (MSE) in Fourier space summed over each time step in the training data:

14 $$\begin{aligned} C = \sum _i \int d^3 \textbf{k}\left| \hat{\textbf{B}}(\textbf{k}, t_i) - \hat{\textbf{B}}_{NN}(\textbf{k}, t_i) \right| ^2, \end{aligned}$$

where $\hat{\textbf{B}}_{NN}$ is the NN prediction for the Fourier transformed magnetic field. By the Plancherel theorem, this quantity is equivalent to the MSE in position space over the training data:

15 $$\begin{aligned} C = \sum _i \int d^3 \textbf{r}\left| \textbf{B}(\textbf{r}, t_i) - \textbf{B}_{NN}(\textbf{r}, t_i) \right| ^2. \end{aligned}$$

To evaluate the model performance at time step *t* we used the relative error of the magnetic field in position space:

16 $$\begin{aligned} \varepsilon (t) = \frac{\int d^3 \textbf{r}|\textbf{B}(\textbf{r},t) - \textbf{B}_{NN}(\textbf{r},t)|}{\int d^3 \textbf{r}|\textbf{B}(\textbf{r},t)|} = \frac{\langle |\textbf{B}- \textbf{B}_{NN}| \rangle }{\langle |\textbf{B}| \rangle }. \end{aligned}$$

Here, $\langle \cdot \rangle$ denotes a spatial integral over the domain. The time averaged $\varepsilon (t)$ was used to evaluate a model over a data set.

## Results

**Figure 6:**
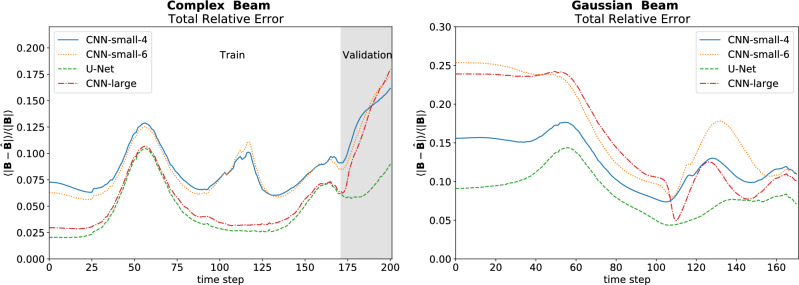
Relative Error of the models on both the complex beam and the Gaussian beam.

**Figure 7:**
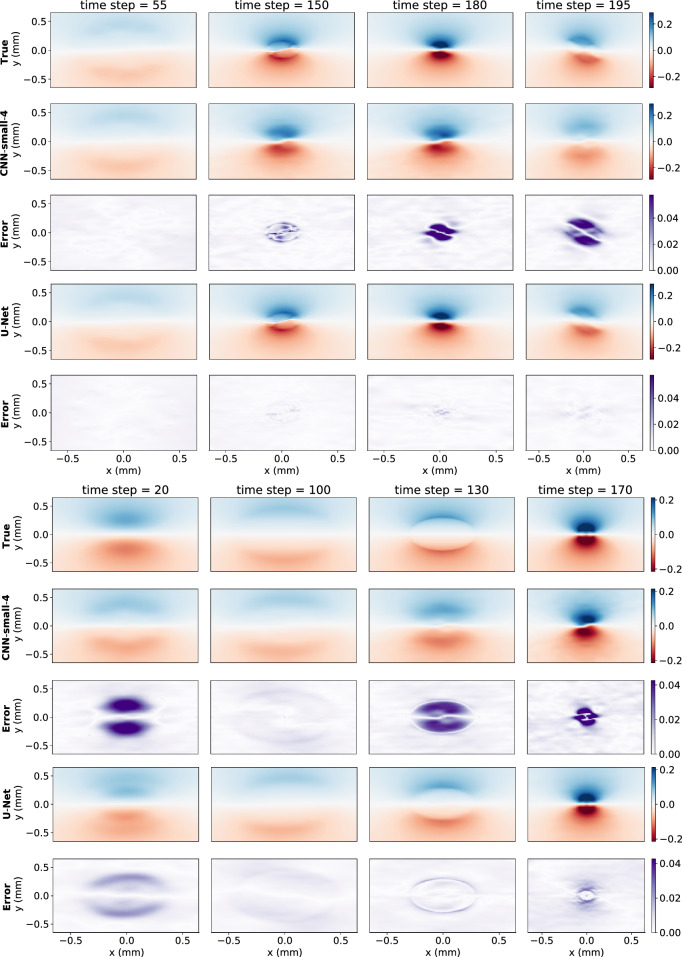
The top 5 rows show model predictions and absolute errors for the complex beam and the bottom 5 rows for the test Gaussian beam for $B_x$ at the $z = 0$ mm slice at various time points. Only the 2 best performing models are shown. The scale of the absolute errors is zoomed in to 1/5 that of the true and model predictions.

The U-Net performed best across all data sets—the training, validation and test data (see Table 2). In addition, as seen in Fig. 6, at each time step for each data set it either had the lowest error or was virtually tied. Remarkably, the U-Net was able to accomplish this with approximately half the training epochs of the smaller CNN’s and a quarter that of CNN-large. Figure 7 shows predictions for $B_x$ at $z = 0$ for the two models which performed best on the test data sets and how they compare to the true values. The results for $B_y$ were similar, and thus aren’t shown.

**Table 2:** Summary of results.

Model	Training error (%)	Validation error (%)	Test error (%)
CNN-small-4	8.2	13.3	12.6
CNN-small-6	7.9	13.5	17.0
U-Net	**4.5**	**6.7**	**8.6**
CNN-large	5.0	12.4	15.9

Error over a data set is $\varepsilon (t)$, defined by Eq. (16), averaged over time. Bold indicates best performing model according to column metric.

The small CNN networks struggled to capture the complexity of the training data. As seen in Fig. 7, while they had a qualitatively correct picture, they struggled on getting many of the finer details correct. The large CNN model was able to capture the training data better, but its predictions quickly deteriorated on the validation set as it got further away from the training data, eventually surpassing the error of CNN-small-4, likely due to overfitting of the larger network. The U-Net captured the training data well and although its error also increases on the validation set, as seen in Fig. 6, it does so at a much smaller rate than any of the CNN models, indicating it learned to generalize better.

All the models struggled the most at the beginning of the Gaussian Beam, where the initial beam was most different from what they were trained on. Of the CNN models, CNN-small-4 did the best job generalizing, though it still lagged behind the U-Net. These results reinforce that the U-Net is doing the best at generalizing.

### Transfer learning

Transfer learning is the process where a model trained on one set of data is retrained on another set. Since much of what was learned earlier can be helpful for the new data set, this cuts down on the training time and is quicker than if one started from scratch. It has been used in cases like Physics-Informed Neural Networks to learn beyond a single instance of a PDE^67^.

**Figure 8:**
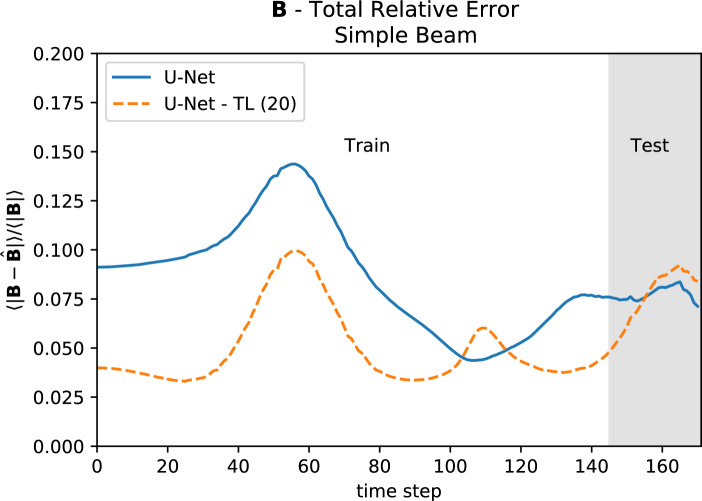
The original U-Net that was trained on part of the complex beam (U-Net) and the model after 20 training epochs on 85% of the Gaussian beam (U-Net-TL(20)).

The U-Net model took 380 training epochs. We took that trained model and retrained it on the first 85% of Gaussian Beam. In only 20 epochs, the model’s relative error on the Gaussian beam dropped dramatically, and its performance was comparable to what it was on the complex beam (see Fig. 8). This demonstrates that our general approach can be useful for a wide range of new beam configurations which the model has not previously seen, so that it can be applied for the study of new different setups.

### Improving generalization

To demonstrate the robustness and generalization capability of our approach when trained with more data, two new datasets were created, complex beam 2 and simple beam 2. Like the previous simulations, it consisted of an electron bunch entering a solenoid field, however now the initial distribution gradually dropped off in the *z* direction, as opposed to suddenly (see Figs. 3 and 4).

**Figure 9:**
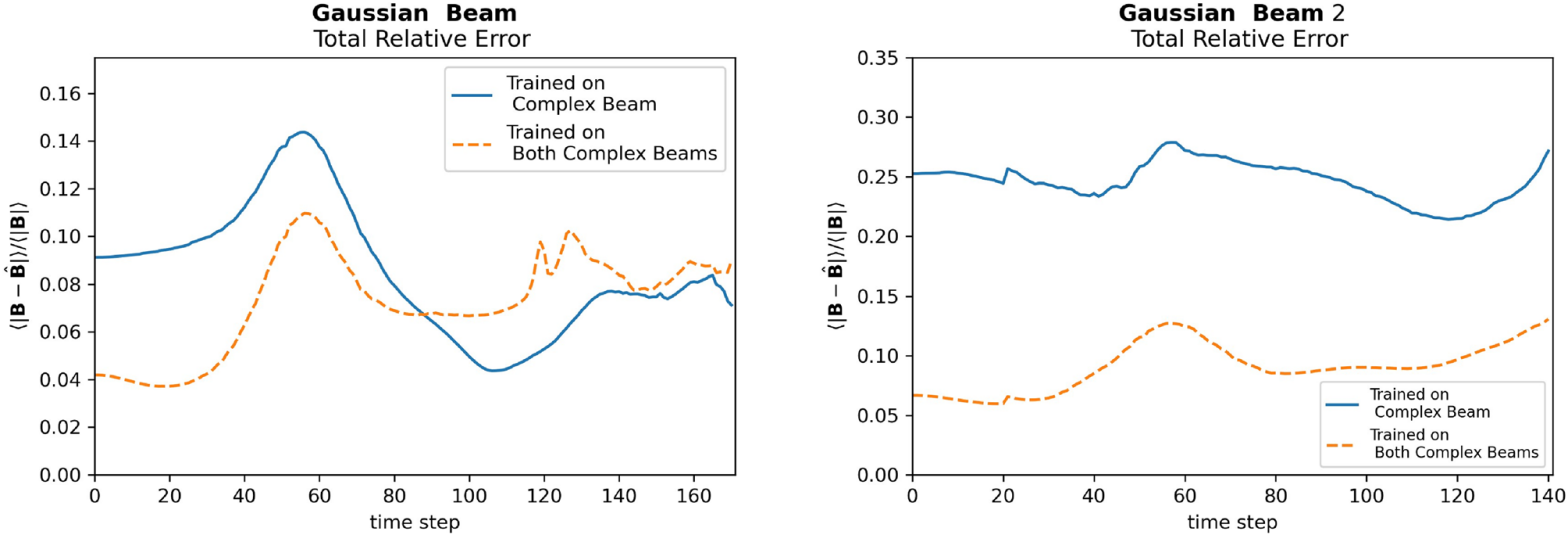
Relative Error of models on the on Gaussian beam and Gaussian beam 2.

Using the pre-trained U-Net from earlier, the network was trained on both complex beam and complex beam 2. The network was tested on both the Gaussian Beam and Gaussian Beam 2, then its performance compared to the network trained on just the complex beam. The results can be seen in Fig. 9. For the Gaussian Beam, the average relative error of the new network on the Gaussian beam was 7.3$\%$, while the network trained exclusively on the complex beam was 8.6$\%$. Thus, the new network improved on predictions for the Gaussian beam with the inclusion of more data in the training set. For Gaussian Beam 2, the network trained on both complex beams consistently performed better, with an average relative error of 9.1$\%$ compared to 24.6$\%$. Thus, we see the inclusion of more data led to an improvement in the network”s ability to generalize.

## Discussion

One reason for the great success of the U-Net could be simply, as mentioned, the skipped connections help with the optimization. Another possibility is that, given that local $J_z$ will correlate strongly with local $\textbf{B}$, the network can start with a good first approximation by not altering the input that much. Indeed, one can compare, for example, $J_z$ at the $z = 0$ mm slice for time step = 150 in the complex beam shown in Fig. 3 with the corresponding magnetic field in Fig. 7. The time of inputting $\hat{J}_{\perp ,z}$ and outputting $\hat{A}_{\perp ,z}$ for the entire test set was of the order of a few seconds. This is in contrast to the GPT simulation, which ran for $\sim$ a day. Of course, the GPT simulator is creating both the matter and electromagnetic fields, so this is not exactly a direct comparison. Nonetheless, it does illustrate some of the potential of the FoHMM-NO method to speed EM simulations greatly.

One way to improve our results would be exposing the model to many distinct simulations during the training phase. This would increase the duration of training, but our results in 5.2 suggests that this will make the model more accurate, robust and would generalize better. There are still some of Maxwell’s equations that are not built in as hard constraints, namely Eq. (8). One possible way to add this as an inductive bias would be to include in the cost function a penalty term that enforces Eq. (8), as is done with physics informed neural networks^61^. Such terms have also been used to enforce the Lorenz gauge for PCNN’s predicting electromagnetic potential fields^40^ and enforcing the mangetohydrodynamics equations with physics informed neural operators^58^. Although this is not a hard constraint, during training it would guide the model to predict more physical solutions. As mentioned, the approach here can be expanded to include $\rho$ and $\textbf{J}$ in the predictions. Alternatively, it can be incorporated into a conventional simulator to speed up it up.

## Conclusions

Using Maxwell’s equation in Fourier space and modeling EM fields via $\hat{A}_{\perp , z}$, we have developed a novel approach to applying machine learning to EM problems. This method has the advantage of incorporating several of Maxwell’s equations as hard constraints, making the output more physically plausible. The FoHM-NO method can be beneficial in computational EM cases where speed is paramount. Here, we exchanged an error level of a few percent for a gain in speed of several orders of magnitude. We found that a U-Net architecture did best both at recreating the fields and generalizing beyond the training data. Given the exact and quantitative nature of physics constraints, domain knowledge can be incorporated into machine learning tools in unique ways. Integrating physics and machine learning in a synergistic way has great potential, but the approaches are still in their infancy. With the advent of powerful new machine learning algorithms, physicists have the opportunity to explore how these new tools can be used to complement or augment more traditional methods to solve previously intractable problems.

## Data Availability

Datasets used and analyzed during the current study are available from the corresponding author upon reasonable request.

## Appendix A: k = 0 case

Taking the Fourier transform of Gauss’s Law for the electric field gives:

17 $$\begin{aligned} \frac{1}{(2\pi )^{3/2}} \int d^3\textbf{r}e^{-i\textbf{k}\cdot \textbf{r}} \nabla \cdot \textbf{E}(\textbf{r}, t) = \frac{\hat{\rho }(\textbf{k}, t)}{\varepsilon _0}. \end{aligned}$$

Integrating the left-hand side by parts:

18 $$\begin{aligned} \lim _{R \rightarrow \infty }\frac{1}{(2\pi )^{3/2}} \int _{S^2(R)} d\textbf{S} \cdot \textbf{E}(\textbf{r}, t) e^{-i\textbf{k}\cdot \textbf{r}} + i\textbf{k}\cdot \hat{\textbf{E}}(\textbf{k}, t)= \frac{\hat{\rho }(\textbf{k}, t)}{\varepsilon _0}, \end{aligned}$$

where $S^2(R)$ means a sphere of radius *R*.

For $\textbf{k}={\textbf {0}}$, $\hat{\rho }({\textbf {0}}, t) = \frac{ Q_{tot}}{(2\pi )^{3/2} }$. The boundary term on the left-hand side of Eq. (18) is a flux, the second term disappears and we get the integral form of Gauss’s law for a large sphere:

19 $$\begin{aligned} \lim _{R \rightarrow \infty }\int _{S^2(R)} d\textbf{S} \cdot \textbf{E}(\textbf{r}, t) = \frac{Q_{tot}}{\varepsilon _0}. \end{aligned}$$

For $\textbf{k}\ne {\textbf {0}}$, the phase inside the boundary term on the left-hand side of Eq. (18) oscillates rapidly because of the large *R*. The boundary term thus disappears and we get the expected expression:

20 $$\begin{aligned} i\textbf{k}\cdot \hat{\textbf{E}}(\textbf{k}, t) = \frac{\hat{\rho }(\textbf{k}, t)}{\varepsilon _0}. \end{aligned}$$

## Appendix B: Longitudinal Ampère–Maxwell

The longitudinal component of Ampère–Maxwell’s equation in Fourier space follows from the continuity equation and Gauss”s law for $\textbf{E}$.

21 $$\begin{aligned} 0&= \frac{\partial \hat{\rho }(\textbf{k}, t)}{\partial t} + i\textbf{k}\cdot \hat{\textbf{J}}(\textbf{k}, t), \nonumber \\ 0&= \frac{\partial \left( \varepsilon _0 i\textbf{k}\cdot \hat{\textbf{E}}(\textbf{k}, t)\right) }{\partial t} + ik \hat{J}_\parallel (\textbf{k}, t), \nonumber \\ 0&= \varepsilon _0 ik \frac{\partial \hat{E}_\parallel (\textbf{k}, t)}{\partial t} + ik \hat{J}_\parallel (\textbf{k}, t), \nonumber \\ 0&= \varepsilon _0 \mu _0\frac{\partial \hat{E}_\parallel (\textbf{k}, t)}{\partial t} + \mu _0 \hat{J}_\parallel (\textbf{k}, t). \nonumber \\ \end{aligned}$$

Since $$\left( i\textbf{k}\times \hat{\textbf{B}}(\textbf{k},t)\right) _\parallel = 0$$ then:

22 $$\begin{aligned} \left( i\textbf{k}\times \hat{\textbf{B}}(\textbf{k},t)\right) _\parallel = \varepsilon _0 \mu _0\frac{\partial \hat{E}_\parallel (\textbf{k}, t)}{\partial t} + \mu _0 \hat{J}_\parallel (\textbf{k}, t). \end{aligned}$$
